# Determinants of Multimorbidity in a Low-Resource Setting: A Population-Based Cross-Sectional Study in Bangladesh

**DOI:** 10.1155/ghe3/2909466

**Published:** 2025-04-04

**Authors:** Syed Toukir Ahmed Noor, Luthful Alahi Kawsar, Mohammad Romel Bhuia

**Affiliations:** Department of Statistics, Shahjalal University of Science & Technology, Sylhet 3114, Bangladesh

**Keywords:** Bangladesh, chronic diseases, low-resource setting, multimorbidity, non-communicable diseases

## Abstract

Multimorbidity is a complex and highly prevalent health condition characterised by the coexistence of two or more chronic diseases within an individual. It is a growing public health issue worldwide, predominantly in low-resource countries like Bangladesh. Therefore, this study aimed to determine the prevalence and associated factors of multimorbidity among the adult population in Bangladesh. A cross-sectional study was carried out among 504 respondents who were 18 years or older. The generalised linear mixed model was used to identify the risk factors. Among the respondents, 65.3% (95% confidence interval [CI]: 61.0 to 69.3) had multimorbidity. The most common chronic conditions were allergic disorder (34%, 95% CI: 30 to 39), gastritis (31%, 95% CI: 27 to 35), low back pain (28.4%, 95% CI: 24.6 to 32.5), oral diseases (27%, 95% CI: 23 to 29) and arthritis (21%, 95% CI: 18 to 25). Middle-aged adults (adjusted odds ratio [AOR] = 7.97; 95% CI: 3.35 to 18.92) and older adults (AOR = 8.44; 95% CI: 1.90 to 36.64) had significantly higher odds of multimorbidity than young adults. Employed respondents had an 86% (AOR = 0.14; 95% CI: 0.07 to 0.36) lower odds of multimorbidity than non-working individuals. Higher sleeping duration (6 to 8 h: AOR = 0.44; 95% CI: 0.25 to 0.80; 8 to 10 h: AOR = 0.26; 95% CI: 0.11 to 0.60), regular vegetable consumption (AOR = 0.42; 95% CI: 0.22 to 0.80) and adequate water intake (AOR = 0.48; 95% CI: 0.29 to 0.79) were protective factors against multimorbidity, whereas obesity increased the odds (AOR = 3.32; 95% CI: 1.06 to 10.43). These findings emphasise the need to promote healthy lifestyle habits, such as maintaining a balanced diet, staying hydrated and engaging in regular physical exercise, to reduce the burden of multimorbidity in low-resource settings.

## 1. Introduction

Multimorbidity is currently one of the major public health problems around the world [1]. Multimorbidity is defined as having more than one (two or more) health conditions in the same individual [2]. It often refers to the existence of multiple chronic diseases concurrently in a person [2]. Multimorbidity has substantial effects on the lives of individuals, their families and societies as well. It is also associated with high and frequent use of healthcare services [3]. The health care costs for frequently treating multiple diseases in a single person are an excessive burden to the overwhelmed health system in low-resource settings like Bangladesh [4]. Annual costs of multimorbidity are also very high in most countries. One of the leading public health agendas of worldwide policymakers and healthcare planners nowadays is to find ways to reduce the burden of multimorbidity [3]. It is, therefore, now a high priority for global health research [5].

The prevalence of multimorbidity varies significantly globally, as highlighted in several systematic reviews and meta-analyses. One study [6] analysed data from 193 countries and reported a wide range of multimorbidity prevalence, from 2.7% to 95.6%, with a pooled global prevalence of 42.4%. Specifically, in low- and middle-income countries (LMICs), the estimated prevalence was 36.8%, while in high-income countries (HICs), it was 44.3%. Similarly, another systematic review and meta-analysis [7], including 70 research conducted in community settings, reported a global prevalence of 31.1%, with higher rates observed in HICs (37.9%) compared to LMICs (29.7%). This study also reported substantial variation in the prevalence of multimorbidity in community settings, ranging from 3.5% in Hong Kong to 70% in Russia. This high prevalence of multimorbidity was responsible for high mortality and substantial healthcare and economic burden globally [7]. Furthermore, a global systematic review and meta-analysis of 126 peer-reviewed studies estimated an overall prevalence of 37.2%, with a prevalence of 38.6% in HICs, 38.7% in upper-middle-income countries (UMICs) and 32.1% in LMICs [8].

Very few studies have been conducted on multimorbidity in Bangladesh. Some of these studies reported very high prevalence, whereas others reported low prevalence. A study conducted on older adults (age > 60 years) in Bangladesh reported that the prevalence of multimorbidity was 56.4%, with 54.1% in men and 64.1% in women [9], whereas another study [10] in Bangladesh reported that 8.4% of people aged 35 years or more have multimorbidity, with 7.7% male and 8.9% female. Moreover, estimates derived using the World Health Surveys (WHS) showed that the prevalence of multimorbidity in Bangladesh was 2.9% in the age group 8–49 years, 10.9% 50–64 years and 12.6% among the elderly aged 65 years [11]. These discrepancies in the reported estimates of multimorbidity in Bangladesh highlight the need for a well-designed population-based study with an adequate and representative sample of the adult population to accurately determine the prevalence of multimorbidity and its associated risk factors.

The prevalence of multimorbidity increases with older age [12]. However, multimorbidity is not only a problem of older age; rather, a high proportion of young adults also suffer from multimorbidity, particularly in low-resource settings, due to a lack of access to adequate healthcare facilities [11]. Furthermore, multimorbidity had a significant impact on the quality of life, as well as healthcare utilisation and outcomes for individuals [13]. Various other factors contributed to the high prevalence of multimorbidity in Bangladesh. Urbanisation, which has led to lifestyle changes and increased exposure to risk factors such as unhealthy diets and physical inactivity, was one of the main contributing factors [14]. Also, poor access to healthcare services in rural areas and low awareness of preventative healthcare are also contributing factors [15].

Thus, epidemiological research on multimorbidity is highly needed to guide prevention and control efforts in low-resource settings, particularly where healthcare resources are scarce. Therefore, this study aims to investigate the prevalence and determinants of multimorbidity in the adult population in a low-resource setting in Bangladesh. The insight into local epidemiology will help reduce the burden of multimorbidity and promote targeted interventions.

## 2. Methodology

### 2.1. Study Design and Setting

This was a cross-sectional study conducted in the Sylhet City Corporation, a northeastern region of Bangladesh, selected as a representative case study for low-resource settings. Primary data were collected between February and April 2023 using a multistage cluster sampling method. Sylhet City Corporation comprises 27 wards and 207 mahallas.

### 2.2. Study Population (Inclusion/Exclusion Criteria)

The target population included all residents aged 18 years or older living in the Sylhet City Corporation of Bangladesh. Only individuals who provided informed consent were included in the study, while those who were not permanent residents or unable to provide consent were excluded.

### 2.3. Sample Size and Sampling

A multistage cluster sampling approach was employed for participant selection. In the first stage, nine wards were selected from the 27 wards using simple random sampling. In the second stage, one mahalla was randomly chosen from each of the selected wards. Since the mahallas were too large to cover entirely, each was divided into compact segments (clusters), each containing approximately 56 adults. In the third stage, a single compact segment was randomly selected from each mahalla, and all adults within the selected segment were included in the study.

Considering the multimorbidity prevalence of 56% in Bangladesh, estimated by Sara et al., 2018 [9], the sample size for this study was calculated using a 95% confidence interval (CI) and a margin of error of 5%. This resulted in an initial required sample size of 379 participants. However, after accounting for a design effect of 1.33 to adjust for cluster sampling, the final sample size was determined to be 504 participants. The detailed calculation can be found in Supporting 1.

### 2.4. Study Variables

#### 2.4.1. Outcome Variable

The outcome variable in this study was the self-reported ‘status of multimorbidity' was the outcome variable of this study. It is a binary variable, categorised as ‘yes' (coded as ‘1') if a participant reported having more than one doctor-diagnosed chronic conditions and classified as ‘no' (coded as ‘0') if they had one or no diagnosed chronic condition.

#### 2.4.2. Case Definition of Multimorbidity

Multimorbidity is commonly defined as the presence of two or more chronic medical conditions in an individual, regardless of whether these conditions are physical, mental or a combination of both [16, 17]. According to the World Health Organisation (WHO), multimorbidity includes conditions that may interact, complicate management and require comprehensive care strategies. These chronic conditions are typically diagnosed by healthcare professionals and confirmed through medical records, though in community-based research, self-reported medical diagnoses are commonly used to establish multimorbidity [16, 17].

In this study, a total of 39 chronic conditions were considered to assess multimorbidity, including allergic disorders, anxiety disorders, arthritis, asthma, atrial fibrillation, attention deficit disorder, bipolar disorder, bronchiectasis, cardiac arrhythmia, cardiomyopathy, cataracts, chronic kidney disease, chronic obstructive pulmonary disease (COPD), constipation, depressive disorder, diabetes mellitus, digestive diseases, eating disorders, eye disease, gastritis, glaucoma, gout, heart failure, haemophilia, hyperlipidaemia, hypertension, ischaemic heart disease, low back pain, low blood pressure, migraine, nausea, neck pain, neurological disorders, oral health issues, osteoporosis, Parkinson's disease, skin disorders, stroke and urinary incontinence.

#### 2.4.3. Explanatory Variables

The study incorporated a wide range of explanatory variables to assess the prevalence and risk factors associated with multimorbidity. These variables spanned several domains, including sociodemographic factors, lifestyle habits and health-related behaviours. The variables included: sex (male, female), age category (young adults [18 to 24 years], mid-adults [25 to 64 years], senior adults [65+ years]), occupation (student/unemployed, business/job holder/labourer, housewife), education level (illiterate, 1 to 9, secondary school certificate (SSC), high school certificate (HSC), bachelor or above), marital status (never married, married, widowed/divorced/separated), family type (joint, nuclear), household income, carer (own self, parents, husband/wife, daughter/son/in-laws or others), tobacco habits (none, cigarette, betel leaf and nut or chewing tobacco), soft drinks consumption (irregular, regular), fast-food consumption (irregular, regular), vegetable consumption (irregular, regular), fruit consumption (irregular, regular), water intake daily (less than eight glasses, eight or more), eggs consumption weekly (no eggs, 1 to 5 eggs, more than five eggs), red meat consumption (never, regularly, occasionally, rarely), physical exercise (no, yes), sleeping hours (4 to 6, 6 to 8, 8 to 10 h or above), childhood trauma (no, yes), BMI (underweight, normal, overweight, obese) and blood pressure at survey time (hypotension, normal, prehypertension, hypertension).

### 2.5. Data Collection Tools

In this study, we employed a combination of tools and techniques to ensure accurate and reliable data collection. A structured quantitative questionnaire was developed to collect self-reported data from the study participants. The questionnaire was pre-tested to ensure clarity, relevance and ease of comprehension, and it included questions covering sociodemographic characteristics, self-reported health conditions and lifestyle factors.

For clinical measurements, we used standardised instruments. Blood pressure was measured using the Omron JPN-500 digital blood pressure monitor, which is known for its reliability and precision in field surveys. Weight was measured using a Premium OTC brand digital weight machine, calibrated before each use to ensure accurate readings.

### 2.6. Statistical Analysis

Exploratory data analysis (EDA) was primarily used to gain insight into the data. Simple descriptive statistics (frequency distribution, mean, median, standard deviations and interquartile range) and graphical representation of background characteristics were used to compare the socioeconomic and demographic conditions of the respondents. Chi-squared test were used to examine the association between the response variable (multimorbidity status) and the set of explanatory variables.

A generalised linear mixed model (GLMM) was used to identify the determinants of multimorbidity. GLMM was used to deal with the dependency in our response variable as data from two or more people living in the same household were correlated. Intra-cluster correlation (ICC) is the term for measuring the correlation between responses among respondents inside the same cluster in a GLMM configuration. The value of ICC ranges from 0 to 1. A GLMM model is only relevant when the ICC is bigger than 0 [18].

The final model included variables that returned a *p* value of less than 0.10 in bivariate analysis. We used the variance inflation factor (VIF) to check the presence of multicollinearity in our fitted model. The variables having VIF values of greater than 5 were excluded from the final fitted model to ensure the absence of multicollinearity in the model.

Adjusted odds ratio (AOR) along with 95% CI were used to present the strength of the risk in the model. The GLMM model was fitted using ‘glmer' function from ‘lme4' package in R (version 4.3.0). All data were analysed with R version 4.3.0. Data entry, coding and editing were done by using STATA V.17.0 (StataCorp, College Station, Texas, USA). This study is reported in accordance with the Strengthening the Reporting of Observational Studies in Epidemiology (STROBE) Statement [19].

### 2.7. Ethical Approval

The principles stated in the Declaration of Helsinki were followed in this research [20]. The Shahjalal University of Science and Technology (SUST) Research Ethics Board, Sylhet-3114, Bangladesh, granted ethical approval for this study (Ethical approval code: SREB/PS/STA/PP 02(2024)).

## 3. Results

### 3.1. Population Characteristics

Among the 504 sampled respondents, 56.8% were women. The majority were Muslim (77.4%) and aged 25 to 64 (67.1%). Occupations included students/unemployed (30.2%), business/job holders/labourers (38.7%) and housewives (31.2%). Most participants were married (65.8%), with 29.6% holding a bachelor's degree or higher. The majority of participants had no tobacco habits (54.6%). Exercise habits varied, with 26% engaging in regular exercise. About half of the respondents had a normal BMI (47.9%), and around 40% were overweight/obese. According to blood pressure at survey time, 42.1% had prehypertension and 19.6% had hypertension (Table 1).

### 3.2. Prevalence of Multimorbidity and Top 15 Diseases

**Figure 1:**
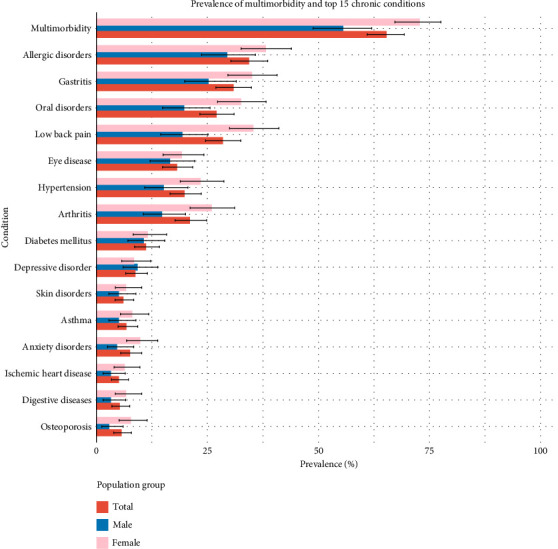
Prevalence of multimorbidity and top 15 chronic conditions of respondents (*n* = 504).

Figure 1 presents the prevalence of multimorbidity and the top 15 chronic conditions across different population groups (Male and Female). The overall prevalence of multimorbidity was 65.3% (95% CI: 61.0, 69.3). Allergic Disorders were the most common condition, affecting 34.3% (95% CI: 30.3, 38.6) of the respondents [38.1% (95% CI: 32.5, 44.0) in females and 29.4% (95% CI: 24.0, 35.6) in males], followed by Gastritis with an overall prevalence of 30.8% (95% CI: 26.9, 34.9) [35.0% (95% CI: 29.7, 40.8) in females and 25.2% (95% CI: 20.1, 31.1) in males]. Low back pain affected 28.4% (95% CI: 24.6, 32.5) of the population, with a prevalence of 35.3% (95% CI: 30.0, 41.1) in females and 19.3% (95% CI: 14.5, 25.1) in males. Oral disorders were present in 27.0% (95% CI: 23.3, 31.0) of individuals [32.5% (95% CI: 27.3, 38.2) in females and 19.7% (95% CI: 14.9, 25.6) in males], while arthritis affected 21.0% (95% CI: 17.7, 24.8) overall [25.9% (95% CI: 21.1, 31.1) in females and 14.7% (95% CI: 10.6, 20.1) in males]. The prevalence of hypertension was 19.8% (95% CI: 16.6, 23.6) [23.4% (95% CI: 18.9, 28.7) in females and 15.1% (95% CI: 11.1, 20.3) in males], and eye disease was reported by 18.1% (95% CI: 14.9, 21.7) of the population [19.2% (95% CI: 15.0, 24.3) in females and 16.5% (95% CI: 12.6, 21.4) in males]. Diabetes mellitus affected 11.1% (95% CI: 8.6, 14.2) of individuals [11.5% (95% CI: 8.4, 15.6) in females and 10.6% (95% CI: 7.0, 15.6) in males], while depression had a prevalence of 8.7% (95% CI: 6.6, 11.5) [8.4% (95% CI: 5.9, 11.9) in females and 9.2% (95% CI: 6.0, 13.8) in males], and anxiety disorders were reported by 7.5% (95% CI: 5.5, 10.2) overall [9.8% (95% CI: 7.0, 13.7) in females and 4.6% (95% CI: 2.8, 7.3) in males]. Asthma was present in 6.7% (95% CI: 4.9, 9.3) [8.0% (95% CI: 5.7, 11.2) in females and 5.0% (95% CI: 3.1, 7.8) in males], while skin disorders were observed in 6.0% (95% CI: 4.2, 8.4) [6.6% (95% CI: 4.5, 9.7) in females and 5.0% (95% CI: 3.1, 7.8) in males]. Osteoporosis affected 5.6% (95% CI: 3.9, 7.9) of the population [7.7% (95% CI: 5.4, 11.0) in females and 2.8% (95% CI: 1.4, 5.5) in males], while digestive diseases and ischaemic heart disease both had a prevalence of 5.2% (95% CI: 3.5, 7.5), with digestive diseases being more common in females [6.6% (95% CI: 4.3, 10.1)] and ischaemic heart disease more prevalent in males [6.0% (95% CI: 3.5, 10.2)]. The prevalence of all self-reported medically diagnosed chronic conditions identified in our study is detailed in Table S1 of Supporting Information. Also, the percentage of top multimorbidities among respondents identified in our study is detailed in Table S2 of Supporting Information.

### 3.3. Prevalence of Multimorbidity by Socioeconomic, Demographic, Food Habits, Behaviours and Health-Related Characteristics

Table 2 presents the distribution of multimorbidity based on socioeconomic and demographic characteristics. The prevalence of multimorbidity was notably higher among women (72.7%) compared to men (55.5%) (p.v = 0.001). Age was also significant, with older adults aged 65 and above showing the highest prevalence (87.2%), followed by middle-aged adults (69.5%) and young adults (47.2%) (p.v = 0.001). Housewives had the highest multimorbidity prevalence (80.3%) compared to other occupational groups (p.v = 0.001). Marital status also influenced multimorbidity, with married individuals reporting a higher prevalence (69.1%) compared to never-married individuals (49.6%) (p.v = 0.001).

Table 3 presents the distribution of multimorbidity based on food habits, behaviours and health-related characteristics. Individuals with irregular vegetable intake had a higher prevalence of multimorbidity (75%) compared to those with regular vegetable intake (62.4%) (*p*=0.012). Similarly, inadequate daily water consumption was associated with a higher prevalence of multimorbidity (71.3%) compared to those with sufficient water intake (55.7%) (p.v = 0.001). Additionally, those who experienced childhood trauma reported a higher prevalence of multimorbidity (80.4%) compared to those with no trauma (63.5%) (*p*=0.018). Furthermore, the prevalence of multimorbidity was higher among overweight (68.9%) and obese (81.8%) individuals compared to those with a normal BMI (62.7%) (*p*=0.006). Finally, individuals with hypertension (76.5%) and hypotension (76.9%) had a close to significantly higher burden of multimorbidity than those with normal blood pressure (*p*=0.050).

### 3.4. Determinants of Multimorbidity in GLMM

**Figure 2:**
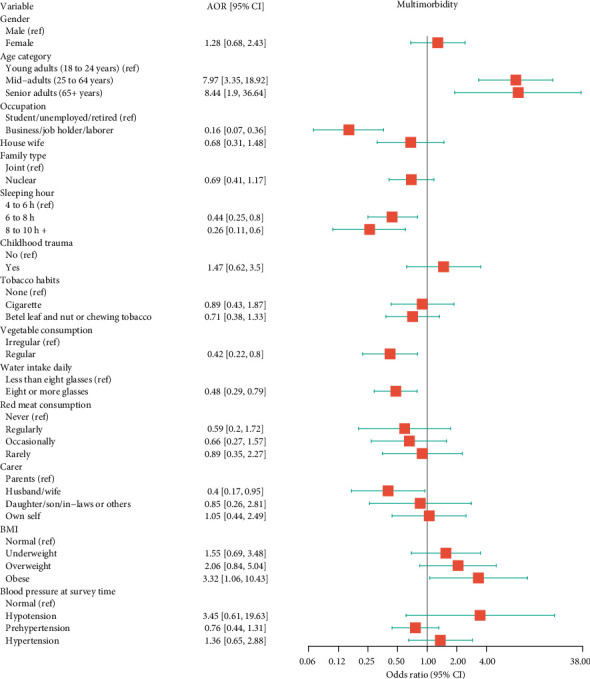
Associated factors of multimorbidity among the adult population in Bangladesh (result from the generalised linear mixed model) (*n* = 504).

The findings from GLMM showed that age, occupation, sleeping hours, vegetable consumption, water intake, carer facility and BMI were important determinants of multimorbidity. Mid-adults were almost eight times more likely to have multimorbidity than young adults (18 to 24 years) (AOR: 7.97; 95% CI: 3.35 to 18.92), while senior adults were 8.44 times more likely (AOR: 8.44; 95% CI: 1.90 to 36.64). Individuals in the workforce (such as businessmen, job holders or labourers) exhibited a significantly lower risk of multimorbidity compared to non-working individuals (such as students, unemployed and retired). Working individuals' odds of multimorbidity were approximately 86% lower (AOR: 0.14; 95% CI: 0.07 to 0.36) than non-working individuals. A higher sleeping duration was also associated with reduced odds of multimorbidity; those who slept for 6 to 8 h daily had a 56% lower risk (AOR: 0.44; 95% CI: 0.25 to 0.80), and individuals who slept for 8 to 10 h daily or more had a 74% lower risk (AOR: 0.26; 95% CI: 0.11 to 0.60) compared to those who slept for only 4 to 6 h daily. Regular consumption of vegetables was a significant protective factor; respondents who regularly consumed vegetables had 58% lower odds (AOR: 0.42; 95% CI: 0.22 to 0.80) than those who did not. Similarly, higher water intake was significantly associated with lower odds of multimorbidity, with those drinking eight or more glasses daily having 52% lower odds (AOR: 0.48; 95% CI: 0.29 to 0.79) than those consuming fewer than eight glasses. Being cared for by a spouse was associated with a reduced risk of multimorbidity; individuals cared for by their spouse had a 60% lower chance (AOR: 0.40; 95% CI: 0.17 to 0.95) compared to those cared for by their parents. Finally, BMI was identified as a significant risk factor for multimorbidity; obese participants had 3.32 times higher odds (AOR: 3.32; 95% CI: 1.06 to 10.43) of having multimorbidity compared to normal-weight individuals (Figure 2). The detailed estimates with 95% CI of these factors are given in Table S3 of Supporting Information.

### 3.5. ICC

According to the ICC estimate, the value found to be 0.129 suggests a moderate level of clustering or similarity in the occurrence of multimorbidity within households in this study. Therefore, the variation between households can explain a 12.9% variation in developing multimorbidity (Table S4).

## 4. Discussion

### 4.1. Main Findings

This study estimated the prevalence of multimorbidity in Bangladesh to be 65.3%, with 55.5% among males and 72.7% among females. The findings of our study align with a global systematic review and meta-analysis of 126 peer-reviewed studies, which similarly reported a higher prevalence of multimorbidity among females (39.4%) compared to males (32.8%) [8]. Moreover, a previous study conducted in Bangladesh also highlighted that women had a higher prevalence of multimorbidity than men [10]. When compared to other LMICs, a systematic review estimated the prevalence of multimorbidity to be 36.8% [6], which is considerably lower than our findings. Similarly, a study conducted in Nepal, a neighbouring country, found the pooled prevalence of multimorbidity to be 25.1%, reflecting potential differences in definitions, study settings or population characteristics [21]. On the other hand, our estimates were consistent with findings from India, another neighbouring country, where the prevalence was reported to be 62.7% [22]. Furthermore, a systematic review focussing on South Asian countries observed a wide variation in the prevalence of multimorbidity, ranging from 4.5% to 83% [23]. These variations highlight the need for more localised studies to better understand the burden of multimorbidity in different contexts. Also, the findings of the GLMM in our study imply that the significant determinants of multimorbidity in Bangladesh (a low-resource setting) were age, occupation, sleeping hours, vegetable consumption, water intake, carer support and BMI.

In this study, respondents' ages were positively associated with a higher chance of developing multimorbidity. As people get older, their immune systems weaken, making them more likely to develop diseases [24]. Smith and colleagues also found a strong relationship between age and the prevalence of multimorbidity, suggesting that age-related physiological changes play a role in the development of multiple chronic diseases [25]. This finding was consistent with many other studies that state that age is a significant risk factor for multimorbidity [26, 27]. In low-income areas, the burden of age-related conditions might be exacerbated due to limited access to healthcare services and inadequate preventive measures [28]. For instance, older individuals in these settings may face challenges accessing routine screenings, medications or health education programs, which increases their vulnerability to developing multiple chronic illnesses [29].

The study also identified obesity as a significant risk factor for multimorbidity. A previous systematic review and meta-analysis study, including 43 studies and nearly one million participants, found that individuals with obesity were significantly more [29] likely to develop multimorbidity compared to those with normal BMI [30]. Additionally, that study also found a dose–response relationship between BMI and multimorbidity, with each 5 kg/m^2^ increase in BMI associated with a 35% higher risk of developing multimorbidity [30]. A few other studies also reported that higher BMI levels have continuously been linked to a higher chance of getting chronic illnesses, such as diabetes, cardiovascular disease and several forms of cancer [31, 32]. The primary reason behind this finding could be that obesity leads to various adverse health effects, such as chronic inflammation, insulin resistance and hormonal imbalances. These conditions create an environment in the body that promotes the development and progression of multiple chronic diseases simultaneously [33]. In low-income areas, the prevalence of obesity may be influenced by urbanisation, limited access to affordable healthy food options and increased reliance on processed and calorie-dense foods [34]. Additionally, the lack of safe recreational spaces or opportunities for physical activity in these regions may contribute to higher rates of obesity and its associated health risks [35].

Occupation was another significant determinant of multimorbidity identified in this study. This study found that people who were in the labour force had a lower risk of multimorbidity than unemployed or retired persons. The result was consistent in several other studies where it was found that multimorbidity was high among individuals who were not in the labour force [36, 37]. On the other hand, there exist few other studies that identified the opposite finding, which was that working people had a higher rate of multimorbidity [38–40]. There could be several reasons behind this finding; perhaps one main reason would be that, depending on the type of work, participating in the labour force frequently involves regular mental and physical stimulation. Numerous health advantages of physical activity include lowering the risk of chronic diseases, including cardiovascular disease, obesity and diabetes [41]. Additionally, cognitively challenging jobs can support the maintenance of brain function and protect against diseases like dementia [42]. In low-income settings, individuals not in the labour force may face additional challenges, such as food insecurity, limited access to healthcare and increased stress due to economic instability, which further exacerbates their risk of multimorbidity.

Our study also observed that sleeping duration played a vital role in reducing adult multimorbidity. It was found that the risk of multimorbidity decreases as the sleeping duration increases (up to 10 h). In line with the result of this study, several studies covering different demography of the world, like China [43], United Kingdom [44], Luxembourg [45], Canada [46], United States [47] and a few other regions, also found that people with short sleeping habits were vulnerable to developing multimorbidity [48, 49]. Lack of sleep damages the immune system and metabolic dysregulation of the body. Numerous physiological functions, such as immunological function, hormone balance and cellular repair, depend on getting enough sleep. Short periods of sleep can interfere with these functions, which can lead to dysregulation and a greater vulnerability to chronic diseases [50]. In addition, increased levels of inflammation in the body have been associated with insufficient sleep. Previous studies have also found that chronic inflammation is a common underlying cause of numerous diseases, such as cardiovascular conditions, diabetes and certain cancers [51]. In low-resource settings, factors such as overcrowded living conditions, noise pollution and increased stress levels may contribute to poor sleep quality, further increasing the burden of multimorbidity.

In this research, regular vegetable consumption was found to be protective against multimorbidity. This finding aligns with other studies that highlight the health benefits of a diet rich in veggies [52, 53]. The WHO suggests consuming at least five servings of fruits and vegetables daily as part of a balanced diet [54]. One possible reason why eating vegetables is linked to a lower risk of multimorbidity is that they are rich in nutrients. Vegetables are an excellent source of dietary fibre, antioxidants, essential vitamins and minerals—all important for preventing chronic illnesses and preserving overall health [55]. However, in low-income areas like Bangladesh, the cost of fresh vegetables, seasonal unavailability and a lack of awareness about the benefits of vegetable consumption may lead to lower consumption rates. Furthermore, reliance on calorie-dense, nutrient-poor diets due to economic constraints could further exacerbate the risk of multimorbidity [56].

Furthermore, adequate water intake was identified as another essential factor of multimorbidity in our research. Adequate hydration is important to prevent various chronic illnesses and maintain good health. The study by Popkin and colleagues brought attention to the significance of consuming enough water for maintaining normal physiological processes and lowering the risk of diseases such as kidney stones, urinary tract infections and constipation [57]. Many previous studies have also found that high water consumption reduces the risk of several diseases [57, 58]. In many low-income areas, limited access to clean and safe drinking water may hinder people from maintaining adequate hydration [59]. Additionally, water scarcity or the prevalence of waterborne diseases may discourage individuals from consuming sufficient amounts of water, further contributing to the risk of developing chronic conditions [60].

This study also found that spouse care plays a vital role in reducing the risk of multimorbidity. Spouse support and care encourage better lifestyle choices, reduce stress, strengthen social connections and boost general well-being [61]. This implies that having a caring partner who offers emotional and practical assistance might help control and avoid multimorbidity. According to an earlier study, spousal support significantly impacts health outcomes [62]. This emphasises the value of social relationships and supports systems in maintaining good health. Also, caregiving and spousal support may reduce stress and enhance mental health, indirectly impacting physical health outcomes. Chronic stress has been associated with a higher chance of developing several chronic diseases, such as metabolic disorders, immune system problems and cardiovascular diseases [63]. Additionally, spousal support can improve social integration and reduce social isolation, both known to be related to adverse health outcomes. Strong social networks and social interaction have been linked to better physical and mental health outcomes, including a lower chance of developing chronic diseases and increased overall well-being [64–66].

### 4.2. Strengths and Limitations of the Study

This study has several strengths that increase the reliability of findings. A comprehensive household survey was conducted in nine distinct wards of a district (Sylhet) in the northeast part of Bangladesh, granting a diverse and representative sample of the low-resource settings. The sample size of 504 individuals ensured the study's statistical power and validity for the larger population. In addition, using a GLMM model rather than a logistic regression model enhances the credibility of the study's findings. Notably, this study reports on more than 40 self-reported medically diagnosed chronic conditions derived from a community-based survey, further contributing to the study's comprehensiveness. Moreover, these estimates will be valuable for future researchers, offering insights and benchmarks for comparative studies and longitudinal analyses.

Despite several strengths, some limitations should be addressed. The reliance on self-reported data introduces the possibility of recall error and social desirability bias. Data collection from healthcare institutions may provide more accurate and reliable estimates. Additionally, the study did not assess the severity of chronic diseases, which could offer a deeper understanding of the complexity of multimorbidity patterns in low-resource settings.

### 4.3. Future Scope

Above all, this work provides a potential foundation for further research and adds valuable knowledge to the field of multimorbidity research in low-resource settings. Future research on multimorbidity may benefit from the use of longitudinal studies, direct data collection from healthcare facilities or medical records and study expansion through the use of a larger, more representative national sample. Also, employing Bayesian GLMM models in future studies can provide valuable insights into the complex interactions between chronic diseases and their collective effects on multimorbidity.

## 5. Conclusion

The prevalence of multimorbidity was significant among the adult population in Bangladesh. Key factors such as age, occupation, sleep duration, vegetable consumption, water intake, spousal support and BMI were found to be associated with multimorbidity. The findings highlight the vital role of sociodemographic and lifestyle variables in preventing and managing multimorbidity. One way to mitigate this risk would be to encourage people to eat vegetables regularly, maintain a sound sleeping habit and drink sufficient water. Addressing occupational influences and encouraging active workforce participation may also be important for prevention. The strong link between obesity and multimorbidity also shows the necessity for effective obesity management. These findings offer valuable guidance for designing targeted interventions and public health strategies to reduce multimorbidity and improve overall health outcomes in low-resource settings like Bangladesh.

## Figures and Tables

**Table 1:** Background characteristics of the respondents.

Variables	Categories	*n* (col %)
Overall		504 (100)

Sex	Male	218 (43.3)
	Female	286 (56.8)

Age category	Young adults (18 to 24 years)	127 (25.2)
	Mid-adults (25 to 64 years)	338 (67.1)
	Senior adults (65+ years)	39 (7.7)

Occupation	Student/unemployed	152 (30.2)
	Business/job holder/labourer	195 (38.7)
	Housewife	157 (31.2)

Education level	Illiterate	82 (16.3)
	1 to 9	120 (23.8)
	SSC	55 (10.9)
	HSC	98 (19.4)
	Bachelor or above	149 (29.6)

Marital status	Never married	137 (27.2)
	Married	331 (65.8)
	Widowed/divorced/separated	35 (7)

Family type	Joint	188 (37.3)
	Nuclear	316 (62.7)

Household income	0–10,000	61 (12.1)
	10,000–15,000	75 (14.9)
	15,000–20,000	84 (16.7)
	20,000–30,000	87 (17.3)
	30,000–40,000	91 (18.1)
	40,000+	106 (21)
	Yes	74 (14.7)

Carer	Own self	181 (36)
	Parents	89 (17.7)
	Husband/wife	170 (33.8)
	Daughter/son/in-laws or others	63 (12.5)
	Satisfied	268 (53.4)
	Not satisfied	31 (6.2)

Tobacco habits	None	275 (54.6)
	Cigarette	71 (14.1)
	Betel leaf and nut or chewing tobacco	144 (27.8)

Soft drinks consumption	Irregular	458 (90.9)
	Regular	46 (9.1)

Fast-food consumption	Irregular	459 (91.1)
	Regular	44 (8.7)

Vegetable consumption	Irregular	116 (23)
	Regular	388 (77)

Fruit consumption	Irregular	333 (66.1)
	Regular	171 (33.9)

Water intake daily	Less than eight glasses	317 (63.2)
	Eight or more	185 (36.9)

Red meat consumption	Never	60 (11.9)
	Regularly	55 (10.9)
	Occasionally	252 (50)
	Rarely	137 (27.2)

Physical exercise	No	373 (74)
	Yes	131 (26)

Sleeping hour	4 to 6 h	185 (36.7)
	6 to 8 h	254 (50.4)
	8 to 10 h or above	65 (12.9)

Childhood trauma	No	447 (88.9)
	Yes	56 (11.1)

BMI	Underweight	59 (11.7)
	Normal	241 (47.9)
	Overweight	148 (29.4)
	Obese	55 (10.9)

Blood pressure at survey time	Hypotension	13 (2.6)
	Normal	179 (35.7)
	Prehypertension	211 (42.1)
	Hypertension	98 (19.6)

**Table 2:** Distribution of socioeconomic and demographic characteristics of the respondents by multimorbidity status (*n* = 504).

Variable	Category	Multimorbidity		*p* value
		No	Yes	
		*n* (%)	*n* (%)	
Overall		175 (34.7)	329 (65.3)	

Sex	Male	97 (44.5)	121 (55.5)	< 0.001^∗^
	Female	78 (27.3)	208 (72.7)	

Age category	Young adults (18 to 24 years)	67 (52.8)	60 (47.2)	< 0.001^∗^
	Mid-adults (25 to 64 years)	103 (30.5)	235 (69.5)	
	Senior adults (65+ years)	5 (12.8)	34 (87.2)	

Occupation	Student/unemployed	51 (33.6)	101 (66.4)	< 0.001^∗^
	Business/job holder/labourer	93 (47.7)	102 (52.3)	
	Housewife	31 (19.7)	126 (80.3)	

Education level	Illiterate	24 (29.3)	58 (70.7)	0.374
	1 to 9	35 (29.2)	85 (70.8)	
	SSC	19 (34.5)	36 (65.5)	
	HSC	39 (39.8)	59 (60.2)	
	Bachelor or above	58 (38.9)	91 (61.1)	

Marital status	Never married	66 (50.4)	65 (49.6)	< 0.001^∗^
	Married	104 (30.9)	233 (69.1)	
	Widowed/divorced/separated	5 (14.3)	30 (85.7)	

Family type	Joint	54 (28.7)	134 (71.3)	0.087
	Nuclear	121 (38.3)	195 (61.7)	

Household income	0–10,000	19 (31.1)	42 (68.9)	0.886
	10,000–15,000	24 (32)	51 (68)	
	15,000–20,000	33 (39.3)	51 (60.7)	
	20,000–30,000	32 (36.8)	55 (63.2)	
	30,000–40,000	32 (35.2)	59 (64.8)	
	40,000+	35 (33)	71 (67)	
	Yes	15 (20.3)	59 (79.7)	

Carer	Own self	42 (47.2)	47 (52.8)	0.001^∗^
	Parents	62 (36.5)	108 (63.5)	
	Husband/wife	9 (14.3)	54 (85.7)	
	Daughter/son/in-laws or others	62 (34.3)	119 (65.8)	
	Satisfied	96 (35.8)	172 (64.2)	
	Not satisfied	12 (38.7)	19 (61.3)	

⁣^∗^*p* < 0.05.

**Table 3:** Distribution of food habits, behaviours and health-related characteristics by multimorbidity status (*n* = 504).

Variable	Category	Multimorbidity		*p* value
		No	Yes	
		*n* (%)	*n* (%)	
Overall		175 (34.7)	329 (65.3)	

Tobacco habits	None	34 (47.9)	37 (52.1)	0.005^∗^
	Cigarette	38 (27.1)	102 (72.9)	
	Betel leaf and nut or chewing tobacco	4 (22.2)	14 (77.8)	

Soft drinks consumption	Irregular	152 (33.2)	306 (66.8)	0.022^∗^
	Regular	23 (50)	23 (50)	

Fast-food consumption	Irregular	158 (34.4)	302 (65.7)	0.326
	Regular	17 (38.6)	27 (61.4)	

Vegetable consumption	Irregular	29 (25)	87 (75)	0.012^∗^
	Regular	146 (37.6)	242 (62.4)	

Fruit consumption	Irregular	109 (32.7)	224 (67.3)	0.192
	Regular	66 (38.6)	105 (61.4)	

Water intake daily	Less than eight glasses	91 (28.7)	226 (71.3)	< 0.001^∗^
	Eight or more	82 (44.3)	103 (55.7)	

Red meat consumption	Never	14 (23.3)	46 (76.7)	0.036^∗^
	Regularly	24 (43.6)	31 (56.4)	
	Occasionally	96 (38.1)	156 (61.9)	
	Rarely	41 (29.9)	96 (70.1)	

Physical exercise	No	133 (35.7)	240 (64.3)	0.341
	Yes	42 (32.1)	89 (67.9)	

Sleeping hour	4 to 6 h	40 (21.6)	145 (78.4)	< 0.001^∗^
	6 to 8 h	101 (39.8)	153 (60.2)	
	8 to 10 h or above	34 (52.3)	31 (47.7)	

Childhood trauma	No	163 (36.5)	284 (63.5)	0.018^∗^
	Yes	11 (19.6)	45 (80.4)	

BMI	Underweight	28 (47.5)	31 (52.5)	0.006^∗^
	Normal	90 (37.3)	151 (62.7)	
	Overweight	46 (31.1)	102 (68.9)	
	Obese	10 (18.2)	45 (81.8)	

Blood pressure at survey time	Hypotension	3 (23.1)	10 (76.9)	0.050
	Normal	66 (36.9)	113 (63.1)	
	Prehypertension	81 (38.4)	130 (61.6)	
	Hypertension	23 (23.5)	75 (76.5)	

⁣^∗^*p* < 0.05.

## Data Availability

The datasets used and/or analysed during the current study are available from the corresponding author upon reasonable request (email at: mr.bhuia@sust.edu, romel_stat@yahoo.com).

## Supporting Information

Supporting Information 1 Supporting 1 method: This document provides detailed information on the sample size calculation for the study. Based on a previous prevalence estimate of 56% for multimorbidity in Bangladesh, we calculated the minimum required sample size using a formula for sample size estimation. After adjusting for a design effect due to cluster sampling, the final sample size was determined to be 504 participants.

Supporting Information 2 Supporting 2 results: This file contains additional analysis results, including the prevalence of self-reported doctor-diagnosed chronic conditions by gender (Table S1), the percentage of top multimorbidities among respondents (Table S2), detailed estimates of the GLMM used to identify the risk factors of multimorbidity (Table S3) and the intra-cluster correlation for the multimorbidity model (Table S4).

## References

[1] Whitty C. J. M., MacEwen C., Goddard A. (2020). Rising to the Challenge of Multimorbidity. *BMJ*.

[2] Johnston M. C., Crilly M., Black C., Prescott G. J., Mercer S. W. (2019). Defining and Measuring Multimorbidity: A Systematic Review of Systematic Reviews. *The European Journal of Public Health*.

[3] Navickas R., Petric V.-K., Feigl A. B., Seychell M. (2016). Multimorbidity: What Do We Know?. *What Should We Do? J Comorb*.

[4] McPhail S. M. (2016). Multimorbidity in Chronic Disease: Impact on Health Care Resources and Costs. *Risk Management and Healthcare Policy*.

[5] Academy of Medical Sciences (2018). Multimorbidity: A Priority for Global Health Research. *Academy of Medical Sciences*.

[6] Ho I. S. S., Azcoaga-Lorenzo A., Akbari A. (2022). Variation in the Estimated Prevalence of Multimorbidity: Systematic Review and Meta-Analysis of 193 International Studies. *BMJ Open*.

[7] Nguyen H., Manolova G., Daskalopoulou C., Vitoratou S., Prince M., Prina A. M. (2019). Prevalence of Multimorbidity in Community Settings: A Systematic Review and Meta-Analysis of Observational Studies. *Journal of Comorbidity*.

[8] Chowdhury S. R., Chandra Das D., Sunna T. C., Beyene J., Hossain A. (2023). Global and Regional Prevalence of Multimorbidity in the Adult Population in Community Settings: A Systematic Review and Meta-Analysis. *EClinicalMedicine*.

[9] Sara H. H., Chowdhury M. A. B., Haque M. A. (2018). Multimorbidity Among Elderly in Bangladesh. *Aging Medicine*.

[10] Khan N., Rahman M., Mitra D., Afsana K. (2019). Prevalence of Multimorbidity Among Bangladeshi Adult Population: A Nationwide Cross-Sectional Study. *BMJ Open*.

[11] Afshar S., Roderick P. J., Kowal P., Dimitrov B. D., Hill A. G. (2015). Multimorbidity and the Inequalities of Global Ageing: A Cross-Sectional Study of 28 Countries Using the World Health Surveys. *BMC Public Health*.

[12] Espeland M. A., Crimmins E. M., Grossardt B. R. (2017). Clinical Trials Targeting Aging and Age-Related Multimorbidity. *Journals of Gerontology-Series A Biological Sciences and Medical Sciences*.

[13] Marengoni A., Angleman S., Melis R. (2011). Aging with Multimorbidity: A Systematic Review of the Literature. *Ageing Research Reviews*.

[14] Moore M., Gould P., Keary B. S. (2003). Global Urbanization and Impact on Health. *International Journal of Hygiene and Environmental Health*.

[15] Rahman F. N., Khan H. T. A., Jahangir Hossain M., Iwuagwu A. O. (2021). Health and Wellbeing of Indigenous Older Adults Living in the Tea Gardens of Bangladesh. *PLoS One*.

[16] Skou S. T., Mair F. S., Fortin M. (2022). Multimorbidity. *Nature Reviews Disease Primers*.

[17] Organization W. H. (2016). *Multimorbidity*.

[18] Merlo J., Chaix B., Yang M., Lynch J., Råstam L. (2005). A Brief Conceptual Tutorial of Multilevel Analysis in Social Epidemiology: Linking the Statistical Concept of Clustering to the Idea of Contextual Phenomenon. *Journal of Epidemiology & Community Health*.

[19] von Elm E., Altman D. G., Egger M., Pocock S. J., Gøtzsche P. C., Vandenbroucke J. P. (2014). The Strengthening the Reporting of Observational Studies in Epidemiology (STROBE) Statement: Guidelines for Reporting Observational Studies. *International Journal of Surgery*.

[20] Williams J. R. (2008). The Declaration of Helsinki and Public Health. *Bulletin of the World Health Organization*.

[21] Sinha A., Suman S. S., Subedi N. (2024). Epidemiology of Multimorbidity in Nepal: A Systematic Review and Meta-Analysis. *Journal of Multimorbidity and Comorbidity*.

[22] Khan M. R., Malik M. A., Akhtar S. N., Yadav S., Patel R. (2022). Multimorbidity and Its Associated Risk Factors Among Older Adults in India. *BMC Public Health*.

[23] Pati S., Swain S., Hussain M. A. (2015). Prevalence and Outcomes of Multimorbidity in South Asia: A Systematic Review. *BMJ Open*.

[24] Weyand C. M., Goronzy J. J. (2016). Aging of the Immune System: Mechanisms and Therapeutic Targets. *Annals of the American Thoracic Society*.

[25] Smith S. M., Wallace E., O’Dowd T., Fortin M. (2021). Interventions for Improving Outcomes in Patients With Multimorbidity in Primary Care and Community Settings. *Cochrane Database of Systematic Reviews*.

[26] Melo L. A. de, Braga L. de C., Leite F. P. P., Bittar B. F., Oséas J. M. de F., Lima K. C. de (2019). Factors Associated With Multimorbidity in the Elderly: an Integrative Literature Review. *Revista Brasileira de Geriatria e Gerontologia*.

[27] Ahmadi B., Alimohammadian M., Yaseri M. (2016). Multimorbidity: Epidemiology and Risk Factors in the Golestan Cohort Study, Iran a Cross-Sectional Analysis. *Medicine*.

[28] McMaughan D. J., Oloruntoba O., Smith M. L. (2020). Socioeconomic Status and Access to Healthcare: Interrelated Drivers for Healthy Aging. *Frontiers in Public Health*.

[29] Bertolazzi A., Quaglia V., Bongelli R. (2024). Barriers and Facilitators to Health Technology Adoption by Older Adults with Chronic Diseases: An Integrative Systematic Review. *BMC Public Health*.

[30] Shan J., Yin R., Panuthai S. (2024). Body Mass Index and Multimorbidity Risk: A Systematic Review and Dose-Response Meta-Analysis. *Archives of Gerontology and Geriatrics*.

[31] Lynch D., Petersen C., Spangler H., Kahkoska A., Batsis J. (2021). Obesity and Multimorbidity in the USA: National Health and Nutrition Examination Surveys 2005-2014. *Innov Aging*.

[32] Lynch D. H., Petersen C. L., Fanous M. M. (2022). The Relationship Between Multimorbidity, Obesity and Functional Impairment in Older Adults. *Journal of the American Geriatrics Society*.

[33] Saltiel A. R., Olefsky J. M. (2017). Inflammatory Mechanisms Linking Obesity and Metabolic Disease. *Journal of Clinical Investigation*.

[34] Andoy-Galvan J. A., Lugova H., Patil S. S. (2020). Income and Obesity in an Urban Poor Community: A Cross-Sectional Study. *F1000Res*.

[35] Ng M., Dai X., Cogen R. M. (2024). National-Level and State-Level Prevalence of Overweight and Obesity Among Children, Adolescents, and Adults in the USA, 1990–2021, and Forecasts up to 2050. *The Lancet*.

[36] Ubalde-Lopez M., Delclos G. L., Benavides F. G., Calvo-Bonacho E., Gimeno D. (2016). Measuring Multimorbidity in a Working Population: The Effect on Incident Sickness Absence. *International Archives of Occupational and Environmental Health*.

[37] Yildiz B., Schuring M., Knoef M. G., Burdorf A. (2020). Chronic Diseases and Multimorbidity Among Unemployed and Employed Persons in the Netherlands: A Register-Based Cross-Sectional Study. *BMJ Open*.

[38] Seo S. (2019). Multimorbidity Development in Working People. *International Journal of Environmental Research and Public Health*.

[39] De Souza A. C. D., Barbosa I. R., de Souza D. L. B. (2021). Prevalence of Multimorbidity and Associated Factors in the Brazilian Working Population. *Revista Brasileira de Medicina do Trabalho*.

[40] Troelstra S. A., Straker L. M., Harris M., Brown S., van der Beek A. J., Coenen P. (2020). Multimorbidity Is Common Among Young Workers and Related to Increased Work Absenteeism and Presenteeism: Results From the Population-Based Raine Study Cohort. *Scandinavian Journal of Work, Environment & Health*.

[41] Brown W. J., Mishra G., Lee C., Bauman A. (2000). Leisure Time Physical Activity in Australian Women: Relationship with Well Being and Symptoms. *Research Quarterly for Exercise & Sport*.

[42] Van Gelder B. M., Tijhuis M. A. R., Kalmijn S., Giampaoli S., Nissinen A., Kromhout D. (2004). Physical Activity in Relation to Cognitive Decline in Elderly Men: The FINE Study. *Neurology*.

[43] Zou C., Sun H., Lu C., Chen W., Guo V. Y. (2022). Nighttime Sleep Duration, Restlessness and Risk of Multimorbidity-A Longitudinal Study Among Middle-Aged and Older Adults in China. *Archives of Gerontology and Geriatrics*.

[44] Sabia S., Dugravot A., Léger D., Hassen C. B., Kivimaki M., Singh-Manoux A. (2022). Association of Sleep Duration at Age 50, 60, and 70 Years With Risk of Multimorbidity in the UK: 25-Year Follow-Up of the Whitehall II Cohort Study. *PLoS Medicine*.

[45] Ruiz-Castell M., Makovski T. T., Bocquet V., Stranges S. (2019). Sleep Duration and Multimorbidity in Luxembourg: Results from the European Health Examination Survey in Luxembourg, 2013-2015. *BMJ Open*.

[46] Nicholson K., Rodrigues R., Anderson K., Wilk P., Guaiana G., Stranges S. (2019). Relationship Between Sleep Patterns and Multimorbidity in the Canadian Longitudinal Study on Aging. *The European Journal of Public Health*.

[47] Krueger P. M., Friedman E. M. (2009). Sleep Duration in the United States: A Cross-Sectional Population-Based Study. *American Journal of Epidemiology*.

[48] von Ruesten A., Weikert C., Fietze I., Boeing H. (2012). Association of Sleep Duration with Chronic Diseases in the European Prospective Investigation into Cancer and Nutrition (Epic)-Potsdam Study. *PLoS One*.

[49] Shi L., Chen S. J., Ma M. Y. (2018). Sleep Disturbances Increase the Risk of Dementia: A Systematic Review and Meta-Analysis. *Sleep Medicine Reviews*.

[50] Haspel J. A., Anafi R., Brown M. K. (2020). Perfect Timing: Circadian Rhythms, Sleep, and Immunity—An NIH Workshop Summary. *JCI Insight*.

[51] Mullington J. M., Haack M., Toth M., Serrador J. M., Meier-Ewert H. K. (2009). Cardiovascular, Inflammatory, and Metabolic Consequences of Sleep Deprivation. *Progress in Cardiovascular Diseases*.

[52] Ruel G., Shi Z., Zhen S. (2014). Association Between Nutrition and the Evolution of Multimorbidity: The Importance of Fruits and Vegetables and Whole Grain Products. *Clinical Nutrition*.

[53] Tanaka S., Yoneoka D., Ishizuka A. (2021). Projections of Disability-Adjusted Life Years for Major Diseases Due to a Change in Vegetable Intake in 2017–2040 in Japan. *BMC Public Health*.

[54] FAO (2021). FAO/WHO International Workshop on Fruits and Vegetables in Preparation for the International Year of Fruits and Vegetables 2021.

[55] Aune D., Giovannucci E., Boffetta P. (2017). Fruit and Vegetable Intake and the Risk of Cardiovascular Disease, Total Cancer and All-Cause Mortality-A Systematic Review and Dose-Response Meta-Analysis of Prospective Studies. *International Journal of Epidemiology*.

[56] Laraia B. A., Leak T. M., Tester J. M., Leung C. W. (2017). Biobehavioral Factors that Shape Nutrition in Low-Income Populations: A Narrative Review. *American Journal of Preventive Medicine*.

[57] Popkin B. M., D’Anci K. E., Rosenberg I. H. (2010). Water, Hydration, and Health. *Nutrition Reviews*.

[58] Serra-Majem L. (2016). Opening Remarks: The Burden of Disease Attributable to Hydration in Europe. *Revista de Neurologia*.

[59] Gomez M., Perdiguero J., Sanz A. (2019). Socioeconomic Factors Affecting Water Access in Rural Areas of Low and Middle Income Countries. *Water (Switzerland)*.

[60] Karamzad N., Safiri S. (2017). A Prospective Study of Water Intake and Subsequent Risk of All-Cause Mortality in a National Cohort: Methodologic Issues. *American Journal of Clinical Nutrition*.

[61] Franks M. M., Shields C. G., Lim E., Sands L. P., Mobley S., Boushey C. J. (2012). I Will if You Will: Similarity in Married Partners’ Readiness to Change Health Risk Behaviors. *Health Education & Behavior*.

[62] Martire L. M., Schulz R., Helgeson V. S., Small B. J., Saghafi E. M. (2010). Review and Meta-Analysis of Couple-Oriented Interventions for Chronic Illness. *Annals of Behavioral Medicine*.

[63] Cohen S., Janicki-Deverts D., Miller G. E. (2007). Psychological Stress and Disease. *JAMA*.

[64] Berkman L. F., Glass T., Brissette I., Seeman T. E. (2000). From Social Integration to Health: Durkheim in the New Millennium. *Social Science & Medicine*.

[65] Holt-Lunstad J., Smith T. B., Layton J. B. (2010). Social Relationships and Mortality Risk: A Meta-Analytic Review. *PLoS Medicine*.

[66] Safwan J., Halat D. H., Akel M. (2023). The Impact of COVID-19 on the Mental Health of Lebanese Pharmacists: A National Cross-Sectional Study. *Frontiers in Public Health*.

