# Supplementary material for: Determinants of Multimorbidity in a Low-Resource Setting: A Population-Based Cross-Sectional Study in Bangladesh
Source: Glob Health Epidemiol Genom. 2025 Apr 4;2025:2909466. doi: 10.1155/ghe3/2909466 (PMC11991861; doi:10.1155/ghe3/2909466)
Supplement: Supporting Information 1 — Supporting 1 method: This document provides detailed information on the sample size calculation for the study. Based on a previous prevalence estimate of 56% for multimorbidity in Bangladesh, we calculated the minimum required sample size using a formula for sample size estimation. After adjusting for a design effect due to cluster sampling, the final sample size was determined to be 504 participants. [file 2909466.f1.docx]

**Supplementary methods**

**Sample Size Calculation:** Considering the multimorbidity prevalence of 56% in Bangladesh, estimated by Sara et al., 2018 ^7^, the minimum required sample size is calculated as follows:

$$n=\frac{z^{2}p(1-p)}{d^{2}}$$

Where 𝑛 = 𝑟𝑒𝑞𝑢𝑖𝑟𝑒𝑑 𝑠𝑎𝑚𝑝𝑙𝑒 𝑠𝑖𝑧𝑒

𝑧 = 𝐶𝑟𝑖𝑡𝑖𝑐𝑎𝑙 𝑣𝑎𝑙𝑢𝑒 𝑓𝑜𝑟 95% 𝑐𝑜𝑛𝑓𝑖𝑑𝑒𝑛𝑐𝑒 𝑖𝑛𝑡𝑒𝑟𝑣𝑎𝑙 =1.96

𝑝 = 𝐸𝑥𝑝𝑒𝑐𝑡𝑒𝑑 𝑝𝑟𝑒𝑣𝑎𝑙𝑒𝑛𝑐𝑒 =0.56 ^7^

𝑑 = 𝑑𝑒𝑠𝑖𝑟𝑒𝑑 𝑎𝑏𝑠𝑜𝑙𝑢𝑡𝑒 𝑝𝑟𝑒𝑐𝑖𝑠𝑖𝑜𝑛=0.05.

Hence,

$$n=\frac{{1.96}^{2}*0.56*0.44}{{0.05}^{2}} \cong379.$$

Considering design effect=1.33 for cluster sampling,

$$n_{adjusted} =379 \times1.33 \cong504.$$

Therefore, data from 504 adults were collected for this study.
