# Supplementary material for: Determinants of Multimorbidity in a Low-Resource Setting: A Population-Based Cross-Sectional Study in Bangladesh
Source: Glob Health Epidemiol Genom. 2025 Apr 4;2025:2909466. doi: 10.1155/ghe3/2909466 (PMC11991861; doi:10.1155/ghe3/2909466)
Supplement: Supporting Information 2 — Supporting 2 results: This file contains additional analysis results, including the prevalence of self-reported doctor-diagnosed chronic conditions by gender (Table S1), the percentage of top multimorbidities among respondents (Table S2), detailed estimates of the GLMM used to identify the risk factors of multimorbidity (Table S3) and the intra-cluster correlation for the multimorbidity model (Table S4). [file 2909466.f2.docx]

**Appendix Results**

Table S1: Prevalence of all self-reported doctor-diagnosed chronic conditions by gender.

| **Chronic condition name** | **Male** | **Female** | **Total** |
| --- | --- | --- | --- |
|  | **% (95% CI)** | **% (95% CI)** | **% (95% CI)** |
| Allergic Disorders | 29.4 (23.7, 35.8) | 38.1 (32.6, 43.9) | 34.3 (30.3, 38.6) |
| Anxiety Disorders | 4.6 (2.5, 8.4) | 9.8 (6.8, 13.8) | 7.5 (5.5, 10.2) |
| Arthritis | 14.7 (10.6, 20.1) | 25.9 (21.1, 31.1) | 21.0 (17.7, 24.8) |
| Asthma | 5.0 (2.8, 8.9) | 8.0 (5.4, 11.8) | 6.7 (4.9, 9.3) |
| Atrial Fibrillation | 0.9 (0.2, 3.6) | 0.3 (0.0, 2.5) | 0.6 (0.2, 1.8) |
| Attention Deficit Disorder | 4.1 (2.2, 7.8) | 2.4 (1.2, 5.1) | 3.2 (2.0, 5.1) |
| Bipolar Disorder | 0.9 (0.2, 3.6) | 2.4 (1.2, 5.1) | 1.8 (0.9, 3.4) |
| Bronchiectasis | 1.8 (0.7, 4.8) | 0.7 (0.2, 2.8) | 1.2 (0.5, 2.6) |
| Cardiac Arrhythmia | 0.5 (0.1, 3.2) | 1.4 (0.5, 3.7) | 1.0 (0.4, 2.4) |
| Cardiomyopathy | - | 0.3 (0.0, 2.5) | 0.2 (0.0, 1.4) |
| Cataracts | 2.3 (1.0, 5.4) | 4.5 (2.6, 7.7) | 3.6 (2.3, 5.6) |
| Chronic Kidney Disease | 1.8 (0.7, 4.8) | 2.4 (1.2, 5.1) | 2.2 (1.2, 3.9) |
| Chronic Obstructive Pulmonary Disease (COPD) | 0.9 (0.2, 3.6) | 0.3 (0.0, 2.5) | 0.6 (0.2, 1.8) |
| Constipation | 2.8 (1.2, 6.0) | 2.4 (1.2, 5.1) | 2.6 (1.5, 4.4) |
| Depressive disorder | 9.2 (6.0, 13.8) | 8.4 (5.7, 12.2) | 8.7 (6.6, 11.5) |
| Diabetes Mellitus | 10.6 (7.1, 15.4) | 11.5 (8.3, 15.8) | 11.1 (8.6, 14.2) |
| Digestive Diseases | 3.2 (1.5, 6.6) | 6.6 (4.3, 10.2) | 5.2 (3.5, 7.5) |
| Eating Disorders | 3.2 (1.5, 6.6) | 6.3 (4.0, 9.8) | 5.0 (3.4, 7.2) |
| Eye Disease | 16.5 (12.1, 22.1) | 19.2 (15.0, 24.2) | 18.1 (14.9, 21.7) |
| Gastritis | 25.2 (19.9, 31.5) | 35.0 (29.6, 40.7) | 30.8 (26.9, 34.9) |
| Glaucoma | 1.4 (0.4, 4.2) | 0.7 (0.2, 2.8) | 1.0 (0.4, 2.4) |
| Gout | - | 0.3 (0.0, 2.5) | 0.2 (0.0, 1.4) |
| Heart Failure | 0.9 (0.2, 3.6) | 0.3 (0.0, 2.5) | 0.6 (0.2, 1.8) |
| Hemophilia | 0.5 (0.1, 3.2) | 1.4 (0.5, 3.7) | 1.0 (0.4, 2.4) |
| Hyperlipidemia | 3.2 (1.5,6.6) | 3.5 (1.9,6.4) | 3.4 (2.1, 5.4) |
| Hypertension | 15.1 (10.9, 20.6) | 23.4 (18.9, 28.7) | 19.8 (16.6, 23.6) |
| Ischemic Heart Disease | 6.0 (3.5, 10.0) | 4.5 (2.6, 7.7) | 5.2 (3.5, 7.5) |
| Low Back Pain | 19.3 (14.5, 25.1) | 35.3 (30.0, 41.1) | 28.4 (24.6, 32.5) |
| Low Blood Pressure | 2.8 (1.2, 6.0) | 7.0 (4.5, 10.6) | 5.2 (3.5, 7.5) |
| Migraine | 0.5 (0.1, 3.2) | 2.8 (1.4, 5.5) | 1.8 (0.9, 3.4) |
| Nausea | 0.9 (0.2, 3.6) | 5.9 (3.7, 9.4) | 3.8 (2.4, 5.8) |
| Neck Pain | 6.9 (4.2, 11.1) | 10.5 (7.4, 14.6) | 8.9 (6.7, 11.8) |
| Neurological Disorders | 1.8 (0.7, 4.8) | 4.2 (2.4, 7.3) | 3.2 (2.0, 5.1) |
| Oral Disorders | 19.7 (14.9, 25.6) | 32.5 (27.3, 38.2) | 27 (23.3,31.0) |
| Osteoporosis | 2.8 (1.2, 6.0) | 7.7 (5.1, 11.4) | 5.6 (3.9, 7.9) |
| Parkinson’s Disease | 0.5 (0.1, 3.2) | 0.3 (0.0, 2.5) | 0.4 (0.1, 1.6) |
| Skin Disorders | 5.0 (2.8, 8.9) | 6.6 (4.3, 10.2) | 6.0 (4.2, 8.4) |
| Stroke | 4.1 (2.2, 7.8) | 1.0 (0.3, 3.2) | 2.4 (1.4, 4.2) |
| Urinary Incontinence | 1.8 (0.7, 4.8) | 4.2 (2.4, 7.3) | 3.2 (2.0, 5.1) |

Table S2: Percentage of top multi-morbidities among respondents

| **Multimorbidity** | **Frequency** | **Percentage** |
| --- | --- | --- |
| Allergic Disorders and Gastritis | 81 | 16.10% |
| Low Back Pain and Gastritis | 69 | 13.70% |
| Allergic Disorders and Low Back Pain | 68 | 13.50% |
| Low Back Pain and Oral Disorders | 66 | 13.10% |
| Gastritis and Oral Disorders | 66 | 13.10% |
| Allergic Disorders and Oral Disorders | 64 | 12.70% |
| Arthritis and Gastritis | 53 | 10.50% |
| Eye Disease and Gastritis | 52 | 10.30% |
| Arthritis and Low Back Pain | 51 | 10.10% |
| Arthritis and Oral Disorders | 48 | 9.50% |
| Allergic Disorders and Arthritis | 43 | 8.50% |
| Low Back Pain and Eye Disease | 43 | 8.50% |
| Eye Disease and Oral Disorders | 41 | 8.10% |
| Allergic Disorders and Eye Disease | 40 | 7.90% |
| Low Back Pain and Hypertension | 39 | 7.70% |
| Arthritis and Hypertension | 38 | 7.50% |
| Gastritis and Hypertension | 38 | 7.50% |
| Allergic Disorders and Hypertension | 37 | 7.30% |
| Diabetes Mellitus and Hypertension | 33 | 6.50% |
| Arthritis and Eye Disease | 31 | 6.20% |
| Hypertension and Oral Disorders | 30 | 6.00% |
| Low Back Pain and Neck Pain | 28 | 5.60% |
| Eye Disease and Hypertension | 27 | 5.40% |
| Gastritis and Neck Pain | 27 | 5.40% |
| Arthritis and Neck Pain | 26 | 5.20% |
| Diabetes Mellitus and Oral Disorders | 26 | 5.20% |
| Allergic Disorders and Neck Pain | 25 | 5.00% |
| Arthritis and Diabetes Mellitus | 25 | 5.00% |
| Allergic Disorders and Diabetes Mellitus | 24 | 4.80% |
| Anemia and Gastritis | 23 | 4.60% |
| Anxiety Disorders and Oral Disorders | 23 | 4.60% |
| Anxiety Disorders and Gastritis | 21 | 4.20% |
| Low Back Pain and Diabetes Mellitus | 21 | 4.20% |
| Neck Pain and Oral Disorders | 21 | 4.20% |
| Diabetes Mellitus and Gastritis | 19 | 3.80% |
| Allergic Disorders and Anemia | 18 | 3.60% |
| Eating Disorders and Oral Disorders | 18 | 3.60% |
| Allergic Disorders and Anxiety Disorders | 17 | 3.40% |
| Allergic Disorders and Depressive Disorder | 17 | 3.40% |
| Digestive Diseases and Oral Disorders | 17 | 3.40% |
| Eating Disorders and Gastritis | 17 | 3.40% |
| Eye Disease and Neck Pain | 17 | 3.40% |
| Hyperlipidemia and Hypertension | 17 | 3.40% |
| Allergic Disorders and Skin Disorders | 16 | 3.20% |
| Arthritis and Eating Disorders | 15 | 3.00% |
| Arthritis and Ischemic Heart Disease | 15 | 3.00% |
| Low Back Pain and Bone Decay | 15 | 3.00% |
| Low Back Pain and Eating Disorders | 15 | 3.00% |
| Allergic Disorders and Asthma | 14 | 2.80% |
| Anemia and Low Back Pain | 14 | 2.80% |

Table S3: Risk factor of multimorbidity using GLMM model.

| **Variable** | **AOR (95% C.I.)** | **p-value** |
| --- | --- | --- |
| **Gender** |  |  |
| Male | 1.00 |  |
| Female | 1.28 (0.68-2.43) | 0.45 |
| **Age category** |  |  |
| Young adults (18 to 24 years) | 1.00 |  |
| Mid-adults (25 to 64 years) | 7.97 (3.35-18.92) | <0.001*** |
| Senior adults (65+ years) | 8.44 (1.90-36.64) | 0.005** |
| **Occupation** |  |  |
| Student / Unemployed | 1.00 |  |
| Business / Job Holder/ Laborer | 0.16 (0.07-0.36) | <0.001*** |
| Housewife | 0.68 (0.31-1.48) | 0.34 |
| **Family Type** |  |  |
| Joint | 1.00 |  |
| Nuclear | 0.69 (0.41-1.17) | 0.17 |
| **Sleeping Hour** |  |  |
| 4 to 6 hours | 1.00 |  |
| 6 to 8 hours | 0.44 (0.25-0.80) | 0.007** |
| 8 to 10 hours + | 0.26 (0.11-0.60) | 0.001** |
| **Childhood Trauma** |  |  |
| No | 1.00 |  |
| Yes | 1.47 (0.62-3.5) | 0.38 |
| **Tobacco habits** |  |  |
| None | 1.00 |  |
| Cigarette | 0.89 (0.43-1.87) | 0.76 |
| Betel leaf and nut or Chewing Tobacco | 0.71 (0.38-1.33) | 0.28 |
| **Vegetable consumption** |  |  |
| Irregular | 1.00 |  |
| Regular | 0.42 (0.22-0.8) | 0.008** |
| **Water intake daily** |  |  |
| Less than eight glasses | 1.00 |  |
| Eight or more glasses | 0.48 (0.29-0.79) | 0.004** |
| **Red meat consumption** |  |  |
| Never | 1.00 |  |
| Regularly | 0.59 (0.2-1.72) | 0.33 |
| Occasionally | 0.66 (0.27-1.57) | 0.34 |
| Rarely | 0.89 (0.35-2.27) | 0.83 |
| **Carer** |  |  |
| Parents | 1.00 |  |
| Husband / Wife | 0.4 (0.17-0.95) | 0.04* |
| Daughter / Son/in-laws or others | 0.85 (0.26-2.81) | 0.79 |
| Own self | 1.05 (0.44-2.49) | 0.91 |
| **BMI** |  |  |
| Normal | 1.00 |  |
| Underweight | 1.55 (0.69-3.48) | 0.29 |
| Overweight | 2.06 (0.84-5.04) | 0.11 |
| Obese | 3.32 (1.06-10.43) | 0.04* |
| **Blood pressure at survey time** |  |  |
| Normal | 1.00 |  |
| Hypotension | 3.45 (0.61-19.63) | 0.16 |
| Prehypertension | 0.76 (0.44-1.31) | 0.32 |
| Hypertension | 1.36 (0.65-2.88) | 0.42 |

*^***^P< 0.001, ^**^ P< 0.01, ^*^ P< 0.05*

Table S4: Intra-cluster correlation of multimorbidity.

| **ICC** | **S.E.** | **95% C.I.** |
| --- | --- | --- |
| 0.129 | 0.031 | 0.068 to 0.190 |
